# Supplementary material for: Temporal recurrence as a general mechanism to explain neural responses in the auditory system
Source: Commun Biol. 2025 Oct 10;8:1456. doi: 10.1038/s42003-025-08858-3 (PMC12514165; doi:10.1038/s42003-025-08858-3)
Supplement: Supplementary file 1 — Supplementary Information [file 42003_2025_8858_MOESM1_ESM.pdf]

Supplementary Note 1: Detrending raw signals with "MedGauss" filter

We demonstrate here the need of detrending neural signals in Wehr and Asari (CRCNS AC1) datasets, consisting of in-vivo patch clamp recordings, akin to drift and noise. We compare in Fig. S1 below the output of a simple linear detrend to our "*MedGauss*" filtering solution, which aims at minimizing manual curation. In this method, we apply a median low-pass filter (2.5 s temporal window) followed by a Gaussian (200 ms of standard deviation) and subtract the output from the raw response:

$$\tilde{r}_{i,j}(t) = r_{i,j}(t) - gauss(med(r_{i,j}(t))) \tag{S1}$$

where $r_{i,j}$ represents the jth response trial to the ith stimulus of a given neuron. The main motivation for this approach is that a single polynomial detrend (as available in common tools such as MATLAB or Scipy) would not be able to correct artifacts of different orders occurring before/after periods of stationarity. This process is illustrated in Supplementary Fig. S1.

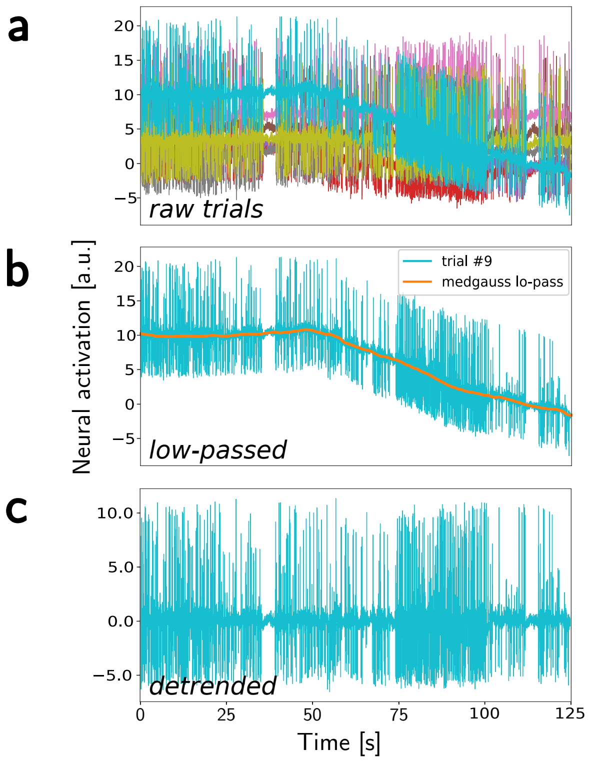

Figure S1. Illustration of the so-called "MedGauss" detrending.(**a**) Response trials of Asari MGB neuron #5 to stimulus #6. They exhibit different temporal means, and some global (i.e., temporally long-range) corruption. (**b**) Response trial of interest and subject to drift, with its low-passed version. (**c**) The detrended signal.

The response trial presented in the above figure shows a spike signal with very nonlinear and long-duration drift. Therefore, it cannot simply be detrended with a linear or even quadratic detrend. Even more generally, polynomial detrend has the inconvenience of requiring the definition of a hyperparameter, the order, which might differ from one recording to another, depending on the type of corruption. Our detrending process can correct global and highly nonlinear drifts of very different shapes, therefore removing the need for manual intervention.

Supplementary Note 2: Connectivity in the first layer of StateNet (ablation study)

We trained the StateNet GRU model on each dataset with fully connected (FC), Locally Connected (LC) and convolutional (CONV) connectivities as the first layer (see Figure S2). The results are reported below in Table S1.

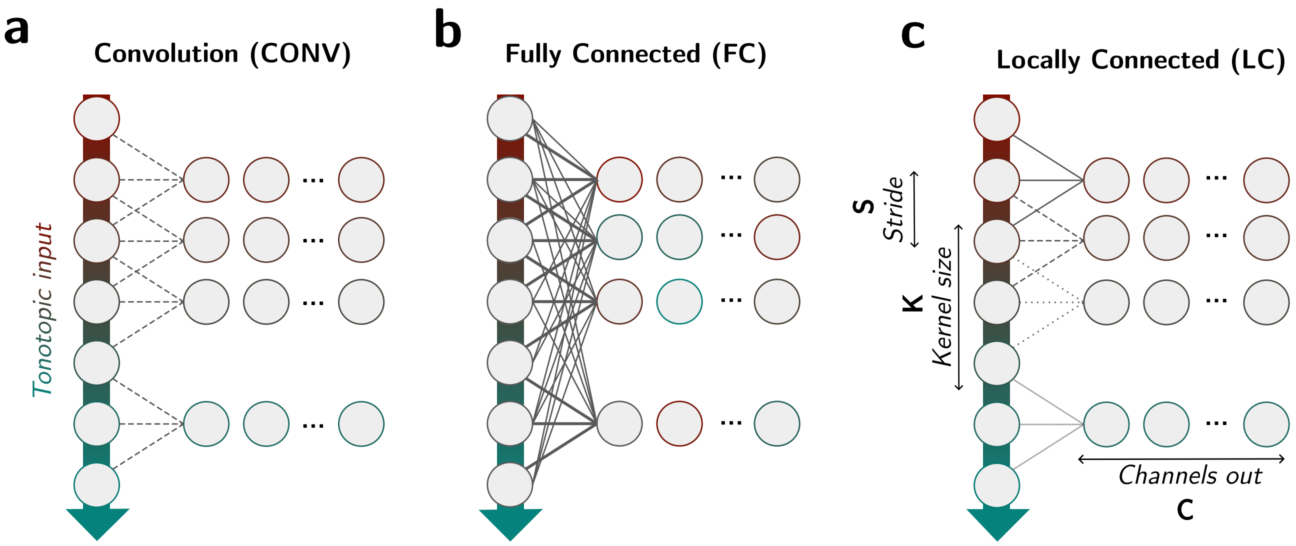

Figure S2. Illustration of the proposed Locally Connected (LC) connectivity compared to Fully Connected (FC) and convolution (CONV). Example proposed for one-dimensional inputs, such as frequency bands, as used in StateNet. In a CONV layer, the same weight kernel is applied at each location of the input, while in an FC layer, each input-output neuron pair has a specific weight. LC is essentially a mix between both: a CONV but without weight sharing, or in other terms, a subset of synaptic connections present in FC.

Dataset		F	K	S	C	# Params			CCnorm [%]		
						FC	LC	CONV	FC	LC	CONV
NS1		34	7	3	7	32,355	30,465	**29,961**	73.8	**75.1**	74.4
NAT4	A1	18	5	2	10	31,241	30,331	**29,971**	**54.7**	54.1	52.5
	PEG	-	-	-	-	-	-	-	64.6	**64.8**	63.3
AA1	MLd	32	7	3	7	26,349	24,774	**24,326**	**74.4**	73.0	69.4
	Field L	-	-	-	-	-	-	-	**71.6**	71.0	69.6
Wehr		49	9	4	5	21,296	19,096	**18,596**	**32.3**	31.0	31.6
Asari	A1	54	-	-	-	25,331	22,631	**22,081**	**23.9**	19.6	23.3
	MGB	-	-	-	-	-	-	-	20.2	20.6	**21.7**

Table S1. Performances of the StateNet GRU model on each dataset with various connectivity schemes for spectral downsampling. Normalized correlation coefficients are given in %, and learnable parameter numbers are given for a backbone with only one output neuron. **Bold font** indicates the best connectivity pattern among the three options available. - symbolizes duplicated entries (see above cell).

As expected, the number of parameters in StateNet-LC models is higher than StateNet-CONV but lower than StateNet-FC (because LC is only a subset of FC). In terms of performance, LC almost always surpasses CONV and yields slightly lower CC_{norm}than FC. Therefore, LC offers an interesting trade-off between neural response fitting accuracy and model size.

Supplementary Note 3: Gradmaps and full dreams for other NS1 neurons

In Figure 3, we showed dreams of one neuron from the NS1 dataset, truncated in the past to focus on the most recent timesteps, for better readability. As a demonstration of the robustness of the approach, we show in Supplementary Fig. S3 that gradmaps of complex StateNet models generalize conventional STRFs for the other neurons of this dataset. This figure also provides another illustration of the temporal extent of context dependence in auditory neurons, as the preferred stimulus (*"dream"*) for this neuron presents complex patterns several seconds prior to current activation in the best-performing StateNet models (GRU, LSTM, S4, Mamba). The complex patterns that have emerged from the optimization procedure in the very long latencies for those models are *not* artifacts: removing them and presenting a truncated version of half the most recent time-steps of their respective dreams to those models leads to lesser activations at the current time (right side of the time axis). Another marking fact from our qualitative analysis of StateNet dreams is the repetition of some warmup patterns (e.g., see GRU on Supplementary Fig. S3), despite the fact that each time-frequency bin was optimized independently from the others (neither structural constraint nor regularization was applied). Finally, this capacity to leverage long-range dependencies is not solely enabled by the model architecture, but also inherited from the neural response fitting process, as a randomly initialized GRU (*"randGRU"*) model displays very short dreams (other neurons not shown).

This Figure also highlights a fading of energy along the borders in both frequency and time dimensions in 2D-CNN GradMaps. This comes inherently from the mode of computation of this network architecture, relying on 2D convolutions with small kernels, favoring the center locations of the input. We do not think that such patterns are present in the brain and therefore identify this as a flaw of this model and a further argument in favor of StateNet models.

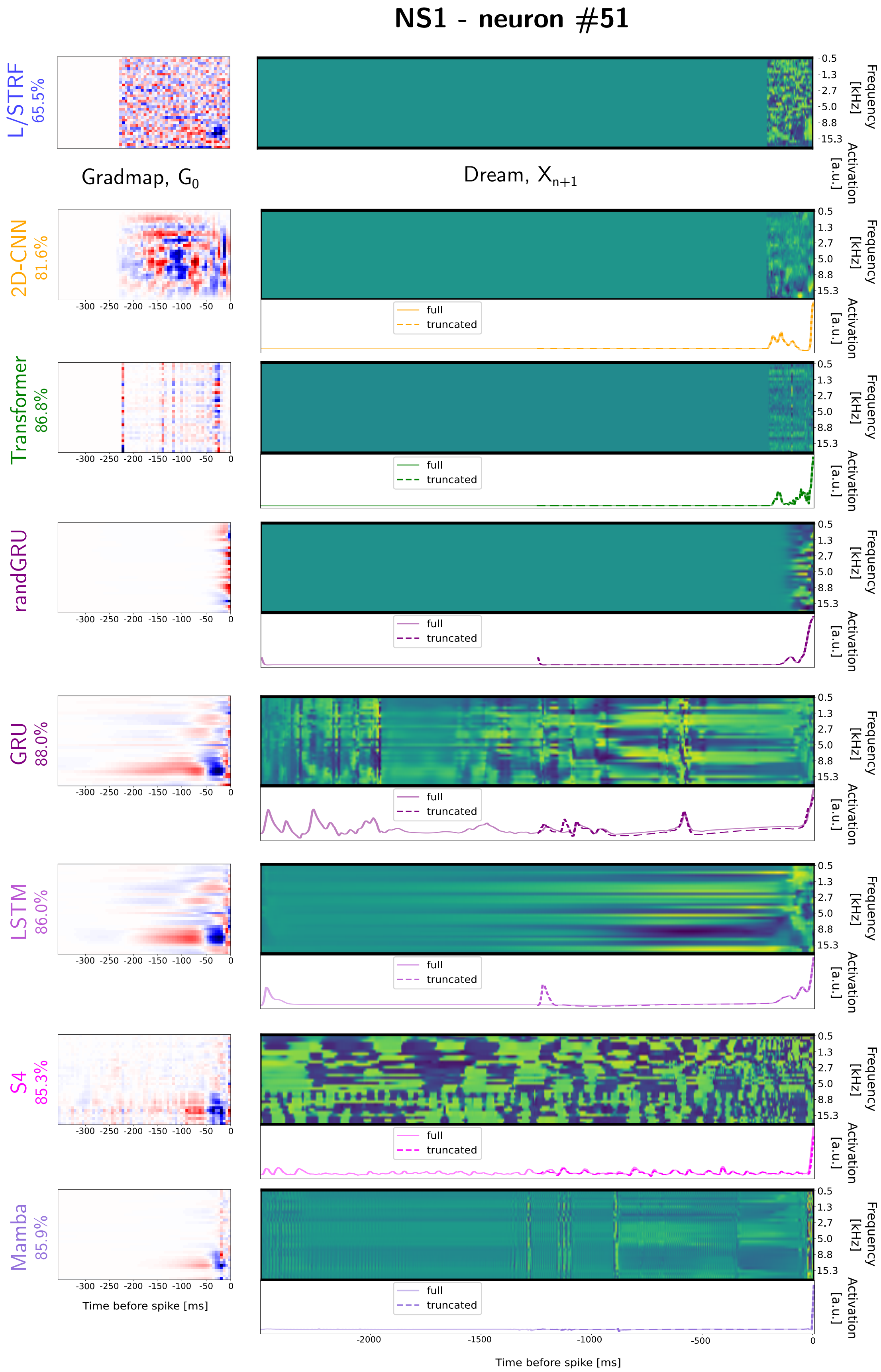

Figure S3. Gradmaps, extended dreams, and responses of other models for another NS1 neuron. *"randGRU"*: randomly initialized and untrained StateNet GRU model. Left of each panel: gradmap g_0. Middle: dream x_{1500}. Bottom: predicted model response to the dream.

Supplementary Note 4: Bridging the gap between STRFs, gradmaps and dreams: theoretical framework

This section aims at consolidating the theoretical relationship between GradMaps, Dreams, and what is commonly referred to as *"linear STRF"* in the literature. This latter term has been used interchangeably as the neuron's "preferred input" eliciting maximal activity[1,2], or as its linear stimulus-response "transfer function"[3–5]. As we will see below, those definitions are related, in particular in the case of the linear (L) model, hence our choice to disentangle "GradMaps" from "Dreams".

Let $\mathcal{M}^*$ be the ideal model of a neuron such that $\mathcal{M}^*(x) = r, \forall x \in \mathbb{R}^{F \times T}$ with $\mathcal{M}^*(x)$ the "true" function implemented by the neuron. Then, the "true" linear STRF of the neuron is defined as the gradmap g_0^* such that:

$$g_0^* = \frac{\partial \mathcal{L}}{\partial x_0} \quad \textbf{(S2)}$$
$$= \frac{\partial - \hat{r}_n(x_0)[T]}{\partial x_0} \quad \textbf{(S3)}$$
$$= \frac{\partial - \mathcal{M}_n^*(x_0)[T]}{\partial x_0} \quad \textbf{(S4)}$$
$$\textbf{(S5)}$$

Thus, when a given model $\mathcal{M}$ tends towards the optimal model $\mathcal{M}^*$ that perfectly captures the function implemented by the neuron on the whole stimulus space, we get:

$$\lim_{\mathcal{M} \to \mathcal{M}^*} - \frac{\partial \mathcal{M}_n(x_0)[T]}{\partial x_0} = g_0^* \quad \textbf{(S6)}$$

It should be reminded that the CC_{norm} metric is only a proxy for the distance separating $\mathcal{M}$ from $\mathcal{M}^*$: it is computed on a subset of stimuli that is limited by experimentation. In any case, as we get better performances at the neural response fitting task with the model $\mathcal{M}$, we can expect experimental STRFs to tend towards the neuron's "true" STRFs.
From our definitions in the Materials and Methods section, and the fact that $x_0 = 0$ is the null vector of the stimulus space $\mathbb{R}^{F \times T}$, the order-1 dream x_1 is:

$$x_1 = x_0 - \alpha g_0 \quad \textbf{(S7)}$$
$$= 0 - \alpha g_0 \quad \textbf{(S8)}$$
$$= -\alpha g_0 \quad \textbf{(S9)}$$
$$= \alpha \frac{\partial \mathcal{M}_n(x_0)[T]}{\partial x_0} \quad \textbf{(S10)}$$

So it turns out that the linear STRF g_0 and the order-1 dream are equal, up to a minus sign and a scaling constant.
The particular case of $\mathcal{M}$ being the Linear model further fueled non-rigorous definitions for the STRF. The L model can be simply written as $\mathcal{M} = Wx + b$, with W and b as the weight matrix and the bias vector. Updating the Eq. S10 yields:

$$x_1 = \alpha \frac{\partial (W x_0 + b)[T]}{\partial x_0} \quad \textbf{(S11)}$$
$$= \alpha W \quad \textbf{(S12)}$$
$$\textbf{(S13)}$$

Then, generalizing to any other dream optimization step i:

$$x_i = x_{i-1} + \alpha \frac{\partial (W x_{i-1} + b)[T]}{\partial x_{i-1}} \quad \textbf{(S14)}$$
$$= x_{i-1} + \alpha W \quad \textbf{(S15)}$$
$$= x_{i-2} + \alpha W + \alpha W \quad \textbf{(S16)}$$
$$= \ldots \quad \textbf{(S17)}$$

It recursively follows that for the L model:

$$x_i = i\alpha W \quad \textbf{(S18)}$$
$$\propto W \quad \textbf{(S19)}$$

We now better understand why the term *"STRF"* has been used to designate both the preferred stimulus of a neuron, as well as changes in stimuli leading to changes in activity[1], or as its convolutional kernel (transfer function): this essentially stems from the linear assumption, and vanishes in our framework, in particular in nonlinear regimes.

Supplementary Note 5: Mathematical mapping of RNN networks to adaptation mechanisms observed in the auditory system

In this paragraph, we demonstrate how deep RNNs can approximate adaptation mechanisms observed in the auditory cortex. We first focus on the gated Recurrent Unit (GRU) because this model leads to the best performance at the neural response fitting task (see Table 1), and then consider other RNN models.

Disclaimer 1: If the nonlinearities in the GRU model prevent it from strictly implementing some adaptation mechanisms, it can still approximate them. In the case where a strict mathematical equivalence is required, working in the linear regime of the σ and $\tanh$ functions can lead to better approximations.

Disclaimer 2: If the demonstrations provided here show that the GRU model can approximate these mechanisms, it does not imply that it is precisely what it learned during the training process. In practice, we can expect that the model learns a mixture of different mechanisms.

Reminder on GRUs equation

The equations of the Gated Recurrent Unit (GRU) are the following:

$$r[t] = \sigma(W_{ir}x[t] + b_{ir} + W_{hr}h[t-1] + b_{hr}) \quad \textbf{(S20)}$$

$$z[t] = \sigma(W_{iz}x[t] + b_{iz} + W_{hz}h[t-1] + b_{hz}) \quad \textbf{(S21)}$$

$$n[t] = \tanh(W_{in}x[t] + b_{in} + r[t] \odot (W_{hn}h[t-1] + b_{hn}) \quad \textbf{(S22)}$$

$$h[t] = (1 - z[t]) \odot n[t] + z[t] \odot h[t-1] \quad \textbf{(S23)}$$

where $\odot$ denotes the element-wise vector product (Hadamard product).

Leaky integration with GRUs

The simplest and most widespread model of neuronal activity is the Leaky-Integrate and Fire (LIF), representing the electric potential of a neuronal membrane viewed as a RC circuit:

$$V[t] = x[t] + (1 - \frac{1}{\tau})V[t-1] \quad \textbf{(S24)}$$

This equation is reminiscent of Eq. S4, when $V = h$ and $z[t] = (1 - \frac{1}{\tau})$. Doing so, $(1 - z[t]) \odot n[t] = \frac{1}{\tau} \odot n[t] \approx x[t]$ in Eq. S4 with $W_{in} = \tau$, $b_{in} = 0$, $r[t] = 0$, $W_{hn} = 0$ and $b_{hn} = 0$. Hence, GRUs can approximate models with an exponential leaky memory, such as the LIF.

Non-leaky integration

GRUs can also implement non-leaky integration, when $z[t] = 1\ \forall t$, e.g. $W_{iz} = W_{hz} = 0$ and b_{iz} or $b_{hz} \gg 0$. It allows the model to remember input patterns over very long periods and to modulate current responses based on a long stimulus history. Because of their vector formulation, some elements of the hidden state can be retained with little or no leak, while others can be more easily replaced by new features. For instance, the first element h_1 of the hidden state can correspond to a permanent memory of some input pattern if $z_1[t] = 1$, while h_2 can be constantly updated if $z_2[t] = 0.2$.
Furthermore, we can construct a GRU model where h_1 has a non-leaky memory (i.e., $z_1[t] = 1$) for most of the inputs, but is still capable of important updates (i.e., $z_1[t] \approx 0$) when encountering specific patterns. This can be achieved by letting $z_1[t]$ depend on $x[t]$ and $h[t-1]$ through non-zero W_{iz} and W_{hz} matrices, as opposed to the previous examples where it was set to a constant leakage rate (related to the time constant τ). As an example, we can imagine a simple system in which z_1 the switch from non-leaky to leaky memory is activated only when the first input component x_1 has high positive values. In the context of audition, it could correspond to a vocalization occurring in a given frequency band.

$$z_1[t] = \sigma(10x_3[t] - 100) \quad \textbf{(S25)}$$

The memory of the h_1 component of the hidden state of such a system only depends on the 3rd input element. With small values of the latter, the update gate is closed, and the current form of h_1 is maintained. However, with large

values of x_3, the update gate opens up and h_1 is changed.

$$x_3[t] = 1 \implies z_1[t] = \sigma(-90) \approx 0 \tag{S26}$$
$$\implies h_1[t] \approx n_1[t-1] \tag{S27}$$
$$x_3[t] = 10 \implies z_1[t] = \sigma(0) = 0.5 \tag{S28}$$
$$\implies h_1[t] = 0.5h_1[t-1] + 0.5n_1[t] \tag{S29}$$
$$x_3[t] = 100 \implies z_1[t] = \sigma(900) \approx 1 \tag{S30}$$
$$\implies h_1[t] \approx h_1[t-1] \tag{S31}$$

Note that this is just an example for illustration. In practice, much more complex patterns of inputs and hidden states are used to open and close the update gate. Thus, gated RNNs such as the GRU can dynamically encode, retain, and forget memory traces of input features in their hidden state over extended numbers of time-steps.

Gain control from amplitude

We showed above that GRUs can dynamically remember or forget stored memory traces based on certain input patterns; one of these patterns could be the input amplitude, in whole or in part. It is indeed widely accepted that sensory neurons adapt their responses based on current or past stimulus amplitude; that is, the mean stimulus level also affects neural gain[6]. In the previous example (Eq. S6-S12), the "key" to control the opening/closing of the update gate was a specific input component (x_3). This behavior could be obtained by setting the weight of this component to a positive value and the weight of other components to zero. But a positive weighted sum of all current input components of $x[t]$ could serve as well as a measure of overall amplitude:

$$z_1[t] = \sigma(\sum_i w_i x_i[t] + b), \quad w_i > 0 \forall i, \quad b \ll 0$$

Note that most cochleagram representations of sound are positive, therefore ridding any potential problems due to negative inputs.

Instantaneous contrast gain control

Another mechanism that has been extensively observed in sensory systems, and notably in the auditory pathway, is *contrast* gain control[6]. It is possible to approximate it from *amplitude* gain control (see above). Instead of using the global input amplitude as a "key", we could rather take some measure of contrast in the current input vector $x[t]$. A valid definition –among others– for this could be the difference in amplitude between a target domain of the stimulus and the remaining "*background*". Taking again the example of speech or animal vocalizations, auditory energy typically falls into a narrow range of relevant frequency bands: humans have, for example, peak sensitivity around 2,000 - 5,000 Hz corresponding to speech[7]. With our proposed definition of contrast, the first component h_1 of the hidden state could be updated depending on the following gate z_1:

$$z_1[t] = \sigma(\sum_i^{i \in voc} w_i x_i[t] - \sum_j^{j \in bkgnd} w_j x_j[t]) \quad w_i > 0 \forall i, \qquad w_j > 0 \forall j$$

Temporal correlation gain control

We showed that GRUs can compute and store (non-)leaky memory traces in their hidden state h, possibly over long sequences. In particular, we showed that gain control can be achieved through constant leak rates, or vary depending on the current stimulus amplitude, in whole or in parts, or on differences in amplitude between some of its parts (i.e., contrast). Sensory neurons can also modulate their responses as a function of the stimulus temporal statistics, like a temporal correlation[8].

Let us define a very simple measure of temporal correlation, say in the first frequency band x_1 of the stimulus x, as an exponential moving average (EMA) of the signal over a recent past, with more weight given to the most recent timesteps. With this definition, stable signals tend to give higher EMAs and noisy signals lower values.

A measure of temporal correlation can therefore be extracted by the GRU as the difference between the current value of the input and its leaky trace.

Implementing AdapTrans with GRUs

AdapTrans is a recently proposed, general descriptive model of auditory ON and OFF responses and adaptation that has been shown to consistently improve neural response fitting performances of stateless models[9]. Applied within each frequency band of the cochleagram, it can be described essentially as computing the weighted difference

between the current signal value and a EMA of the latter in a recent past. In other terms, it is an IIR filter that can be defined with the following recursive equation:

$$y_{ON}[n] = x[n] - (a + w - aw) \times x[n-1] + a \times y_{ON}[n-1]$$

From the demonstrations provided above, we can show that GRUs can also approximate AdapTrans. Indeed, GRUs can compute an EMA of an input stimulus feature x_1 with a certain time constant and store it into a hidden state element, for example h_2. z_2 can be chosen to reflect the exponential time constant (AdapTrans parameter a). At the current time-step t, this stored EMA value can be used to compute a weighted difference (AdapTrans parameter w) with the value of the current input $x_1[t]$. AdapTrans is already a very general model of adaptation that encompasses prior approaches like IC adaptation[10]. Thus, as GRUs are capable of approximating AdapTrans, they are capable of approximating these more specific models too.

The strength that prior models of adaptation do not have

What essentially makes GRUs so powerful is their vector formulation, allowing them to compute and store both global and local features at the same time, for later use. These intermediate calculations enable very elaborate gain control mechanisms. In comparison, AdapTrans, IC adaptation, and STP[11] rules only work frequency-wise and only *within* each spectral band. Prior studies have suggested that different frequency bands have indeed different adaptation parameters, but this alone might not be sufficient to truly reflect the behavior of actual neurons. Instead, we can see in our results that taking into account the contributions from neighboring and distant frequencies with RNNs in vector formulation allows better fits to neuronal responses. This is consistent with the idea that neurons modulate their dynamics from signal statistics outside of their receptive fields[6].

Capabilities of other RNN models

We demonstrated that GRUs have the computational power to implement, or at least approximate, a wide variety of functions that have been extensively observed in biological systems, and notably in the auditory pathway. These demonstrations were performed on the GRU because this model leads to the best performances at the neural response fitting task in our study. Nonetheless, the same demonstrations hold for LSTMs because these models are also gated RNNs but with more equations and parameters, and as such, can reproduce the functions performed by the GRUs.

On the other hand, the Elman (vanilla) RNN models are capable of implementing a constant –null or positive– leak, but quickly encounter some limitations, precisely because their leak is fixed and cannot depend on spectro-temporal stimulus statistics or the hidden state itself. As a reminder, the Elman RNNs are defined by:

$$h[t+1] = \tanh(W_{ih}x[t] + b_{ih} + W_{hh}h[t-1] + b_{hh})$$

Therefore, contrast gain control or temporal correlation gain control are not achievable with these architectures, which could explain their lesser performances compared to the family of gated RNNs.

Supplementary Note 6: Dataset and model details

The L, LN, NRF, DNet and Transformer model used a full spectro-temporal integration window spanning the F frequency bins of the spectrograms (different for each dataset, see table below) and T time bins (T was changed in Fig. 2 and took values of 1, 5, 11, 21, 41). For the 2D-CNN model, the temporal integration window was controlled by varying the temporal size K_t of convolution kernels, taking values of 1, 3, 5, 9, and 15. Each corresponded to temporal integration windows T of respectively 1, 7, 13, 25, and 43. Hyperparameters controlling the hidden size followed similar values to our previous study and were fixed across datasets[12]; these values were selected close to their original publication. Specifically, NRF and DNet models had 20 hidden units. The three convolutions of the 2D-CNN model had 10 output channels, and after flattening, the penultimate fully connected layer had 90 hidden units. The architectural hyperparameters for the StateNet models are contained in supplementary content *"connectivity in the first layer of StateNet"*.

			Datasets						
			NS1	CRCNS AC1		NAT4		CRCNS AA1	
				Wehr	Asari	PEG	A1	MLd	Field L
**Literature**		original paper	Harper et al.[13]	Asari et al.[14]	Asari et al.[14]	Pennington and David[15]		Theunissen et al.[16]	
		others	Rahman et al.[17]	Machens et al.[4]	Asari and Zador[18]	Pennington and David[19]		Singh and Theunissen[20]	
**Recordings**		animal model	ferret	rat		ferret		zebra finch	
		brain areas	A1, AAF	A1	A1 / MGB	PEG	A1	MLd	Field L
		signal type	extracellular	intracellular		extracellular		extracellular	
		# valid neurons	73	20	35 / 11	777	339	50	50
**Stimuli**		duration	5 s	7.5 - 15 s	2 - 7 s	1 s		1-5 s	
		# sounds	20	from 3 up to 63		577 (training), 18 (test)		20	
		# repeats	20	from 1 up to 25		1 (training), 20 (test)		10	
		time bins	5 ms	5 ms	5 ms	10 ms		1 ms	
		# frequency bins	34	49	54	18		32	
**Models**	L & LN	#params	37 - 1,397 (+4)	52 - 2012 (+4)	57 - 2,217 (+4)	18 - 741 (+4)		35 - 1315 (+4)	
	NRF & DNet	n_hidden	20						
		#params	765 - 2,7965 (+21)	1,065 - 40,265 (+21)	1165 - 44,635 (+21)	445 - 14,845 (+21)		725 - 26,325 (+21)	
	2D-CNN	Kf	6	9		3		6	
		c_hidden	10						
		n_hidden	90						
		#params	18,635 - 36,275	24,665 - 51,125	29,165 - 55,625	11,705 - 20,525		16,835 - 34,475	
	Transformer	embedding_dims	48						
		n_heads	4						
		n_layers	2						
		#params	27,285 - 29,205	24,665 - 29,925	28,245 - 30,165	26,517 - 28,437		27,189 - 29,109	
	StateNet	K	7	9		5		7	
		S	3	4		2		3	
		C	7	5		10		7	
		# params (RNN)	10,585	6,776	7,991	10,451		8,646	
		# params (GRU)	30,465	19,096	22,631	30,331		24,774	
		# params (LSTM)	40,405	25,256	29,951	40,271		32,838	
		# params (S4)	28,645	20,966	23,471	28,511		24,900	
		# params (Mamba)	39,145	25,966	29,831	39,011		32,334	
**Training**		population fitting	yes	no		yes		yes	
		batch size	1	1		16		1	

Table S2. Review of the datasets used in this study. Number of parameters for stateless models and DNet are a minimum and a maximum, depending on their explicit integration window size. (+4) correspond to the 4 additional learnable parameters introduced by the output nonlinearity of the LN model. (+21) correspond to the additional learnable parameters introduced by the learnable time constants of the leak of the DNet model. StateNet models (proposed) are shaded in gray.
