# Supplementary material for: Temporal recurrence as a general mechanism to explain neural responses in the auditory system
Source: Commun Biol. 2025 Oct 10;8:1456. doi: 10.1038/s42003-025-08858-3 (PMC12514165; doi:10.1038/s42003-025-08858-3)
Supplement: Supplementary file 2 — Description of Additional Supplementary Files [file 42003_2025_8858_MOESM2_ESM.docx]

Description of Additional Supplementary Files

**File name:** Supplementary Data 1

**Description:** Numerical data for the main figures.
